# Vitamin D Supplementation Is Associated with Inflammation Amelioration and Cognitive Improvement in Decompensated Patients with Cirrhosis

**DOI:** 10.3390/nu17020226

**Published:** 2025-01-09

**Authors:** Raquel Diaz-Ruiz, Maria Poca, Eva Roman, Rocio Panadero-Gomez, Berta Cuyàs, Irene Bañares, Angela Morales, Marta Puerto, Rocio Lopez-Esteban, Elena Blazquez, Marta Fernández-Castillo, Rafael Correa-Rocha, Marta Rapado-Castro, Irene Breton, Rafael Bañares, German Soriano, Rita Garcia-Martinez

**Affiliations:** 1 Department of Digestive Diseases, Instituto de Investigación Sanitaria, Hospital General Universitario Gregorio Marañón, Universidad Complutense Madrid, 28007 Madrid, Spain; diaz.ruiz.r@gmail.com (R.D.-R.); marpucan@yahoo.es (M.P.); rbanares@ucm.es (R.B.); 2 Centro de Investigación Biomédica en Red de Enfermedades Hepáticas y Digestivas (CIBERehd), 28029 Madrid, Spain; mpoca@santpau.cat (M.P.); eroman@santpau.cat (E.R.); bcuyas@santpau.cat (B.C.); gsoriano@santpau.cat (G.S.); 3 Department of Gastroenterology, Hospital de la Santa Creu i Sant Pau, Institut de Recerca Sant Pau (IR Sant Pau), Universitat Autònoma de Barcelona, 08041 Bellaterra, Spain; 4 Department of Child and Adolescent Psychiatry, Institute of Psychiatry and Mental Health, Instituto de Investigación Sanitaria, Hospital General Universitario Gregorio Marañon, Universidad Complutense Madrid, Centro de Investigación Biomédica en Red-Salud Mental (CIBERSam), 28009 Madrid, Spain; r.panaderogomez@gmail.com (R.P.-G.); mrapado@iisgm.com (M.R.-C.); 5 Instituto de Investigación Sanitaria Gregorio Marañon, 28009 Madrid, Spain; banares.irene@googlemail.com; 6 Nutrition Unit, Hospital General Universitario Gregorio Marañon, Universidad Complutense Madrid, 28007 Madrid, Spain; apmoralesc@gmail.com (A.M.); irenebreton@gmail.com (I.B.); 7 Laboratory of Immune-Regulation, Instituto de Investigacion Sanitaria Gregorio Marañon, 28009 Madrid, Spain; rocio.lopez@iisgm.com (R.L.-E.); eblazquez@hggm.es (E.B.); marta.fernandez@iisgm.com (M.F.-C.); rafael.correa@iisgm.com (R.C.-R.); 8 Department of Psychiatry, The University of Melbourne, Melbourne, VIC 3053, Australia; 9 Department of Internal Medicine, Instituto de Investigación Sanitaria, Hospital General Universitario Gregorio Marañon, Universidad Complutense Madrid, 28007 Madrid, Spain

**Keywords:** vitamin D, cognitive function, liver cirrhosis, inflammation, human

## Abstract

**Background/Objectives:** Decompensated cirrhosis is characterized by systemic inflammation and innate and adaptive immune dysfunction. Hepatic encephalopathy (HE) is a prevalent and debilitating condition characterized by cognitive disturbances in which ammonia and inflammation play a synergistic pathogenic role. Extraskeletal functions of vitamin D include immunomodulation, and its deficiency has been implicated in immune dysfunction and different forms of cognitive impairment. The aim was to assess changes in cognitive function and inflammation in decompensated patients with cirrhosis receiving vitamin D supplementation. **Methods**: Patients with cirrhosis discharged from decompensation in two tertiary hospitals in Spain (from September 2017 to January 2020) were assessed before, at 6 and 12 months after vitamin D supplementation. A comprehensive neuropsychological battery and neuroinflammatory markers were examined. In a subgroup of patients, peripheral immune blood cells were analyzed. **Results**: Thirty-nine patients were recruited. Of those, 27 completed the 6 months evaluation and were analyzed [age 62.4 ± 11.3 years; 22 men; Model for End-Stage Liver Disease (MELD) 11.7 ± 4.0; prior overt HE 33%; median 25-hydroxyvitamin D (25OHD) plasma level 12.7 µgr/L] and 22 achieved 12 months assessment. At baseline, learning and memory (R = 0.382; *p* = 0.049) and working memory (R = 0.503; *p* = 0.047) subtests correlated with plasma 25OHD levels. In addition, processing speed (R = −0.42; *p* = 0.04), attention (R = −0.48; *p* = 0.04), Tinnetti balance (R = −0.656; *p* < 0.001) and Tinnetti score (R = −0.659; *p* < 0.001) were linked to neuroinflammation marker IL-1β. Patients with lower 25OHD had a greater proportion of TH1cells at baseline and a larger amelioration of IL-1β and IL-6 following supplementation. An improvement in working memory was found after 25OHD replacement (46.7 ± 13 to 50 ± 11; *p* = 0.047). **Conclusions**: This study supports that vitamin D supplementation modulates low-grade inflammation in decompensated cirrhosis providing cognitive benefits, particularly in working memory.

## 1. Introduction

Hepatic encephalopathy is a brain dysfunction caused by liver failure and/or the presence of portosystemic shunts [1]. Overt (clinically manifested) HE occurs in up to 40% of patients with cirrhosis while covert HE develops in up to 80% of them [2]. Apart from its high prevalence, the clinical expression of neuropsychiatric manifestations results in a highly debilitating condition that severely affects patients’ autonomy and their caregivers [2]. Also, covert HE has been associated with impaired daily functioning and fitness to drive [3] and an increased risk of falls by up to x10 [4]. Its pathogenesis is still poorly understood, in which ammonia was classically considered a key factor; remarkably, inflammation plays a synergistic role with ammonia in HE development [5].

Several studies have previously shown that cirrhosis-associated immune dysfunction (CAID) is a hallmark of cirrhosis, participating in decompensation and contributing to poor outcomes [6,7,8,9,10,11]. Cirrhosis-associated immune dysfunction is a dynamic alteration characterized by systemic inflammation and innate and adaptive immune deficiency with different intensities depending on the cirrhosis stage. The low-grade systemic inflammatory phenotype is characterized by the production of pro-inflammatory cytokines [7], the expression of activation antigens in circulating immune cells [8], TH1 polarization [9,10] and B cells activation [11] and is present in patients with cirrhosis without organ failure. This fact is relevant since CAID (even low-grade phenotypes) contributes to the clinical course of cirrhosis, driving acute decompensation and outcomes such as acute kidney injury (AKI) [12] and overt HE [5,13].

Malnutrition is a common issue in decompensated cirrhosis, occurring in 20–50% of patients, mirroring the severity of liver failure [14]. In this context, vitamin D deficiency has been reported in more than 80% of patients evaluated for liver transplant [15]; moreover, its intensity parallels liver dysfunction and is associated with the risk of bacterial infection and mortality [16,17,18,19]. Vitamin D has gained interest in the last decades because of its pleiotropic extraskeletal functions. Indeed, there is evidence indicating that hypovitaminosis D is a predisposing risk factor for, among others, cognitive impairment [20,21] and cardiovascular and musculoskeletal disorders. In fact, in a large community-based sample, low 25OHD was associated with poorer neuropsychological function and smaller hippocampal volume [20]. Also, hypovitaminosis D was found to be associated with all-cause dementia in a cohort of elder patients receiving home services [21]. In addition, the vast majority of the immune system cells express the vitamin D receptor [22]. In vitro studies exposed the immunomodulatory properties of vitamin D. So, it was observed that vitamin D changes T-Lymphocytes towards a downregulation in pro-inflammatory Thelper-1 and the promotion of the Therlper-2 cells (more tolerogenic phenotype) [22,23].

Very little information is available regarding the potential relationship between vitamin D and HE in decompensated cirrhosis. Vidot retrospectively described in 2017 an association between moderate/severe vitamin D deficiency and overt HE [24]. More recently other cross-sectional studies found differences in 25OHD among grades of overt HE [25,26,27]. Furthermore, it has been identified that vitamin D deficiency was also associated with the presence of covert HE, the development of overt HE and mortality [28]. However, neither causality nor a mechanism for this association has been demonstrated.

Despite the lack of robust evidence of the beneficial effect of vitamin D supplementation in patients with cirrhosis, recent guidelines find it reasonable to assess and treat vitamin D deficiency in this setting [14,29]. Thus, it is conceivable that vitamin D supplementation could potentially exert anti-inflammatory effects improving CAID and HE.

This prospective study investigated the dynamic changes in cognitive function and inflammation in decompensated patients with cirrhosis who received vitamin D supplementation following discharge from acute decompensation.

## 2. Materials and Methods

### 2.1. Design

This is an observational prospective study performed in decompensated patients with cirrhosis in two tertiary hospitals in Spain (Hospital General Universitario Gregorio Marañon—HGUGM, Madrid and Hospital Santa Creu i Sant Pau—HSCSP, Barcelona) between September 2017 and January 2020. Consecutive patients discharged after an acute decompensation episode and screened for nutritional status and treated according to local practice [30,31] were assessed before initiating vitamin D supplementation and then at 6 and 12 months after enrolment. The study was approved by the Institutional Review Board and Ethics Committee of HGUGM (protocol code SUPRAVID on 21 March 2017) and HSCSP (26 June 2017) and all patients signed written consent for participation.

### 2.2. Patients

Decompensated patients with cirrhosis discharged after hospitalization at both institutions were screened for participation. They were invited to participate if the following features were present: (i) decompensated cirrhosis (as clinically manifested ascites, variceal bleeding, HE, AKI-type hepatorenal syndrome [32]) (ii) discharge from liver disease decompensation in the previous 6 weeks; and (iii) age > 18 years. Exclusion criteria were as follows: (i) human immunodeficiency virus infection; (ii) hepatocellular carcinoma beyond Milan criteria; (iii) comorbidities with life expectancy < 6 months; (iv) vitamin D supplementation already prescribed at the time of evaluation; (v) contraindication for vitamin D supplementation; (vi) neurological conditions preventing the assessment of cognitive function (dementia, cerebrovascular disease, …); (vii) previous liver transplantation; (viii) recent (last 6 months) harmful use of alcohol (men > 40 g/d, women > 25 g/d); (ix) cholestatic liver disease; (x) treatment with direct antiviral agents for hepatitis C within the last 6 months; (xi) Grade 3 acute-on-chronic liver failure (ACLF) at the time of evaluation; (xii) MELD > 30; (xiii) immunosuppressive therapy.

Baseline visits were scheduled after acceptance at intervals ranging between 1 and 6 weeks. History of liver disease, complications of cirrhosis and medical treatment were recorded at baseline and during the follow-up. Any clinical event, including infections, was specifically recorded; additionally, neurological assessment, conventional blood test and plasma aliquots for additional analysis were performed in every visit (baseline, 6 and 12 months). Patients recruited in HGUGM also gave samples for peripheral blood immune cell assessment.

### 2.3. Treatment

Vitamin D status was evaluated by plasma levels of 25OHD according to local laboratory procedures. Those patients with insufficiency (25OHD 20–30 ng/mL or 50–75 nmol/L) or deficiency (25OHD < 20 ng/mL or <50 nmol/L)) of vitamin D were supplemented according to the local guidelines available at the time of inclusion and supervised by a nutrition expert in each institution. Briefly, those patients with sufficient vitamin D levels did not receive supplements, those who had insufficiency received Calcifediol 0.266 mg (16,000 UI; Hidroferol^®^, Faes Farma, Leioa, Spain) every two weeks and those with deficiency received 0.266 mg every week with regular controls and readjustments (more detailed information in Supplementary Paragraph S1). In case other nutritional deficits were detected, they were also supplemented (Supplementary Table S1).

### 2.4. Neuropsychological Assessment

Cognitive function was evaluated at baseline, 6 and 12 months using a comprehensive neuropsychological approach designed to assess cognitive alterations described in HE. This battery included tests that assessed processing speed, executive function, visuo-motor abilities, learning and memory, working memory and attention [33] providing they had available Spanish normative values. Additionally, we used the short battery Psychometric Hepatic Encephalopathy Score (PHES). Parallel alternated versions of the same test were used when available (i.e., HVLT) for the follow-up assessments. All patients were alert, oriented and without flapping when tests were performed.

A detailed explanation of the neuropsychological testing is provided in Supplementary Table S2. Briefly, the evaluation included the following:

- Visuo-motor abilities: Grooved Pegboard test, WAIS IV Block Design subtest, the PHES subtests serial dotting test (SDT) and line drawing test (LDT).
- Executive function: the PHES subtests number connection test A (NCT-A) and B (NCT-B) and the Stroop Test interference score.
- Processing speed: the PHES subtest digit symbol test (DST).
- Attention: the Stroop Colour and Word scores from the Stroop test.
- Working memory: The WAIS IV Letter-Number sequencing subtest.
- Learning and memory: The Hopkins verbal learning test.

The neuropsychological assessments were conducted by a trained specialist at each centre. The evaluations were performed in the same environment conditions. The raw scores for each test were transformed into T-scores, following the formula T-score = 50 + 10([x-x_n_]/SD_n_), in which x is the raw result of the test, x_n_ is the mean value and SD_n_ is the standard deviation of the test in the normal population. Normative values were available from Spanish population data, adjusted by age, sex and years of education. In case the given data were a scaled value, then the T value was obtained following the formula T = 16.667 + 3.333 * SP in which SP is the scaled value. The test scores were grouped into cognitive indexes for each cognitive domain as follows: *processing speed* (digit symbol test), *executive function* (NCT-A, NCT-B and Stroop Test interference score), *visuomotor function* (WAIS IV Block design, Grooved, SDT and LDT), *learning and memory* (Hopkins verbal learning test), *working memory* (WAIS IV Letter-Number sequencing subtest) and *attention* (Stroop Test Words and colours total scores). An overall score (*global cognitive index*) was calculated as the average of all the subtests. T-Score always has a mean of 50 and an SD of 10. The definition of impairment was based on the T values: 30–40 (mild), 20–29 (moderate) and <20 (severe) according to standard proceedings [34]. Covert HE was defined as PHES < −4.

### 2.5. Falls Assessment

- Fall definition and quantification. Falls were defined using the World Health Organization definition as follows: “A fall is an event which results in a person coming to rest inadvertently on the ground or floor or other lower level”. Number of falls within the year before inclusion was recorded at the baseline visit and specifically recorded on every visit during the whole period of the study. The retrospective and prospective number of falls was estimated monthly.
- Risk fall assessment. Risk falls were evaluated using the Tinetti balance and gait assessment [35]. Scores ≤ 18, between 19 and 23 and ≥24 indicated high, moderate or low risk of falls, respectively.

### 2.6. Fractures Assessment

- Risk of fractures was calculated using the Fracture Risk assessment tool (FRAX) score with the Spanish reference population to calculate the percentage risk at 10 years of osteoporotic fracture and hip fracture based on demographic and clinical characteristics of patients and femoral neck bone mineral density when available (https://frax.shef.ac.uk/FRAX/tool.aspx?lang=en, 2 November 2024).

### 2.7. Infections Assessment

Similar to falls assessment, clinically evident infections (as diagnosed by a physician) were recorded and quantified retrospectively (within the year before the inclusion) and then prospectively.

### 2.8. Inflammatory Markers

- *Serum concentrations of inflammatory mediators*: Frozen aliquots of plasma were used for the determination of certain markers previously known to be involved in neuroinflammation (CCL2-MCP1—Chemokine system CC ligand2-Monocyte chemotactin protein 1; IL-12p70—Interleukin12-; IL-1β—Interleukin 1beta; GM-CSF—Granulocyte Macrophage Colony-Stimulating Factor; TNF-α 2nd gen—Tumour necrosis factor alpha-; IL-6—2nd gen-Interleukin 6; CX3CL1/FRACTALKINE—Chemokine (C-X3-C motif) ligand 1-) along with vitamin D binding protein (VITDBP) and hepcidin levels. Customized Simple-Plex immunoassay kits (Bio-techne) were used according to the manufacturer’s instructions (https://www.bio-techne.com/reagents/simple-plex-immunoassays/assay-menu?pdfSource=true_neuroinflammation-assay-brochure#human, 2 November 2024).
- *Peripheral blood cell populations*: Fresh peripheral blood samples were obtained only from the subgroup of patients included in HGUGM (n = 14) because they must be freshly processed (within the first 18 h from extraction). Whole blood samples were labelled for surface markers with the fluorochrome-labelled antibodies distributed in three cytometry panels to detect several subsets of innate and adaptive cells, in addition to their differentiation and activation status, following the already published protocols [36]. Whole blood was stained with an antibody mix for each panel, and after incubation, red blood cells were lysed using the TQ-Prep Workstation (Beckman Coulter). After the lysis, stained blood was analyzed by flow cytometry (Gallios; Beckman Coulter, Villepinte, France). Absolute count (cells per µL of blood) was determined using Flow-Count Fluorespheres (Beckman Coulter) and results were processed by the software Kaluza (version 2.3, Beckman Coulter).

### 2.9. Statistical Analysis

Continuous variables were reported as mean ± standard deviation or median (inter-quartile range -IQR-) as appropriate. A Chi-square test or Fisher’s exact test was used to study the existence of significant differences between nominal variables. Normality of continuous variables was explored using Shapiro–Wilk Test. Depending on the variable’s distribution, parametric or non-parametric tests were applied to study the differences between groups of patients (Student’s *t*-test, the Mann–Whitney Rank Sum test or Kruskal–Wallis test) or intrasubjects (Paired-samples *t*-test, repeated-measures ANOVA, Wilcoxon test or Friedman test). Pearson’s or Spearman’s correlation coefficient tests were applied to study correlations between variables. The Jonckheere–Terpstra test was applied to evaluate the association between variables with ordered categories. A *p* value < 0.05 was considered statistically significant. The statistical calculations were performed with SPSS 20.0 software (SPSS, Chicago, IL, USA).

## 3. Results

**Figure 1:**
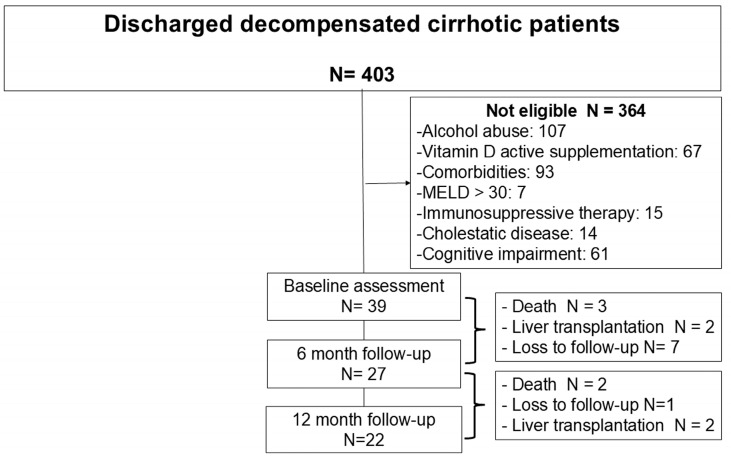
Flowchart of patients evaluated for participation.

During the inclusion period, 403 patients were evaluated for inclusion/exclusion criteria at the time of hospital discharge. Three hundred and sixty-four were not eligible (alcohol abuse n = 107; vitamin D supplementation n = 67; comorbidities n = 93; MELD > 30 n = 7; immunosuppressive therapy n = 15, cholestatic disease n = 14; cognitive impairment n = 61). Thirty-nine patients were finally included. Twenty-seven and twenty-two patients completed the 6-month and 12-month evaluation, respectively (Figure 1).

The patient’s baseline characteristics are summarized in Table 1. Twelve patients (44.4%) had infections in the year before hospital admission, all requiring hospitalization. All subjects had at least one hospitalization in the year before admission, with the most frequent causes being decompensation ascites (n = 25) and overt HE (n = 12). Regarding vitamin D status, 8 patients had insufficiency and 19 had deficiency. Median baseline 25OHD was 12.7 µg/L [Q1 = 8.8 and Q3 = 21]. Patients from HSCSP were older with better liver function (MELD 9.2 ± 2.5 vs. 14.0 ± 3.8, *p* = 0.001) and those from HGUGM were more likely to suffer non-SBP infections. Vitamin D supplementation was initiated at the inclusion visit in all cases.

### 3.1. Baseline Evaluation

#### 3.1.1. Cognitive Function

Cognitive evaluation showed mild disturbances in attention and visuomotor function (Table 2) that were not obvious in clinical evaluation since none of the patients exhibited overt HE.

According to PHES, six patients (22%) had covert HE. Plasma levels of 25OHD did not show any association with cognitive indexes (Table 3).

**Figure 2:**
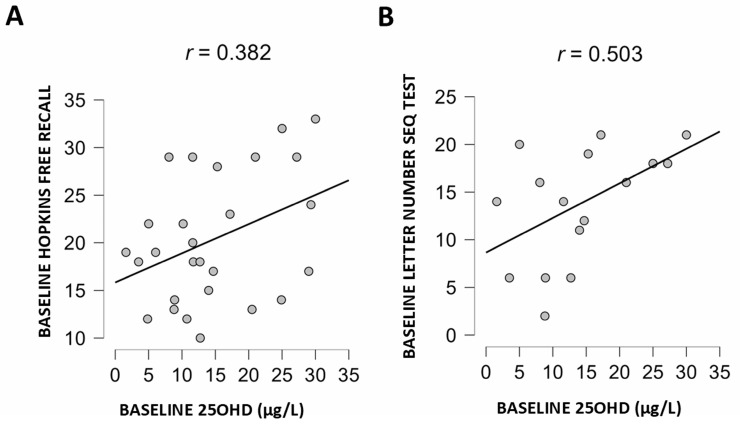
Association between baseline levels of 25OHD and neuropsychological test. (**A**) Association with Hopkins free recall (Pearson’s test), (**B**) association with WAIS IV Letter-Number sequencing subtest (Spearman’s test).

However, there was a correlation with the learning and memory Hopkins free recall subtest (R = 0.382; *p* = 0.049, Figure 2A) and with the working memory WAIS IV letters-numbers sequencing test (R = 0.503; *p* = 0.047, Figure 2B). In addition, there was an association between some cognitive indexes and markers of inflammation (Table 3). Specifically, higher plasma IL-1β levels were associated with worse processing speed (R = −0.42; *p* = 0.04) and attention (R = −0.48; *p* = 0.04).

#### 3.1.2. Assessment of Risk Falls and Fracture Risks

At inclusion, eleven patients (41%) reported falls within the last year, with a mean of 0.1 ± 0.2 falls per patient and month (Table 2). There was no association between the number of previous falls, Tinnetti scale and FRAX with baseline 25OHD.

Interestingly, an association between the Tinnetti balance (R = −0.682; *p* = 0.001) and the Tinnetti score (R = −0.647; *p* = 0.002) with inflammatory markers was observed. Thus, the higher the plasma levels of IL1β, the greater the risk of falls.

#### 3.1.3. Infections

Twelve patients reported infections that required hospitalization within the year before inclusion (0.064 ± 0.09 infection per patient and month, Table 1).

#### 3.1.4. Inflammation

At baseline evaluation, patients were stable without signs of clinically significant systemic inflammation or infection. They also had normal plasma C-reactive protein (CRP) without evidence of organ failure, a phenotype not consistent with high-grade systemic inflammation. We did not find any association between neuroinflammatory markers and 25OHD (Supplementary Table S3).

**Figure 3:**
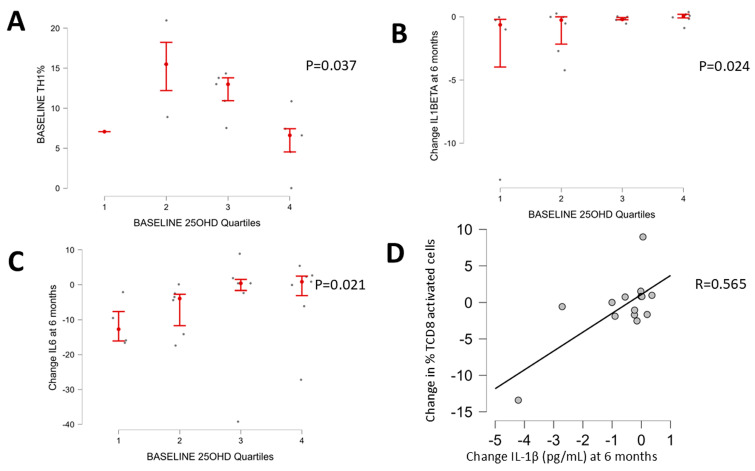
Association between 25OHD and inflammation at baseline and during the follow-up. (**A**) Association between baseline plasma levels of 25OHD and percentage of TH1 cells (Jonckheere–Terpstra, *p* = 0.037) showing that the higher the baseline plasma 25OHD levels, the lower the percentage of TH1 cells in peripheral blood. Decrease at 6 months from baseline in Il-1β (**B**) and IL-6 (**C**) according to the baseline 25OHD quartile (Jonckheere–Terpstra, *p* < 0.05). Positive correlation between the decrease in the percentage of TCD8-activated cells at 6 months and the decrease in plasma levels of IL 1β (R = 0.565; *p* = 0.035, (**D**)).

In the subgroup of patients in which we assessed the distribution of peripheral blood immune cells according to the baseline plasma 25OHD (Supplementary Table S3), we found a negative correlation between 25OHD and the percentage of TH1 cells (the higher the baseline 25OHD levels, the lower the TH1 response, Figure 3A).

### 3.2. Follow-Up Evaluation

The adherence to vitamin D supplementation was very high since the compliance was 89% at 6 months and 95% at 12 months. A significant increase in plasma 25OHD was observed at 6 and 12 months (baseline 14.6 ± 9.0 µg/L vs. 6 months 37.4 ± 20.4 µg/L vs. 12 months 42.6 ± 18.5 µg/L; *p* < 0.001) without significant adverse events. Indeed, 63% of patients (n = 17) achieved sufficiency and 22% remained deficient at 6 months (Table 4). Also, a significant improvement in cholesterol, prealbumin and vitamin A was observed. A raise in hemoglobin levels was also noticed at 6 months. No relevant changes in liver function tests were observed.

#### 3.2.1. Cognitive Function

At 6 months, patients also exhibited mild alterations in visuomotor and attention domains. Compared to the baseline, a significant improvement in working memory (baseline 46.7 ± 13 vs. 6 months 50 ± 11; *p* = 0.047, Table 2) and a trend towards improvement in processing speed (*p* = 0.051) and executive functions (*p* = 0.07) were noticed. No correlations between cognitive changes with baseline 25OHD, changes in vitamin D status, baseline inflammatory markers or changes in inflammatory markers were found. During the follow-up only one to three patients were taking benzodiazepines or antidepressants (Table 4) and we did not find an association between this treatment and cognitive function. Regarding comorbidities, patients with type 2 diabetes on oral hypoglycaemic drugs (n = 10) showed similar cognitive scores at baseline but exhibited a greater improvement in executive function at 6 months (7.2 [20.3] vs. 0.6 [6.7] *p* = 0.023). Despite this different evolution, no significant differences in cognitive scores at 6 and 12 months between diabetic and non-diabetic patients were observed.

#### 3.2.2. Assessment of Falls and Fracture Risks

Fifteen falls in 10 patients (37%) were reported during the follow-up, which represents a mean of 0.050 ± 0.07 falls per patient and month (Table 2). No significant changes in the fall risk assessment or fracture risk were found.

#### 3.2.3. Infections

The mean number of infections during follow-up was 0.055 ± 0.10 per patient and month without significant differences with the incidence observed before inclusion (*p* = 0.68; Table 4). We did not find any association between vitamin D status at baseline and the incidence of infections.

#### 3.2.4. Inflammation

A significant amelioration in inflammatory mediators was observed during the study (Table 4). There was a decrease in CCL2MCP1 (298 ± 136 pg/mL vs. 240 ± 100 pg/mL; *p* = 0.026), IL-1β (1.9 ± 3.7 pg/mL vs. 0.4 ± 0.4 pg/mL; *p* = 0.026) and IL-6 (13.3 ± 10.6 pg/mL vs. 9.6 ± 8.6 pg/mL; *p* = 0.037) at six months. Additionally, a decrease in hepcidine was detected (baseline 37,532 ± 35,265 pg/mL vs. 6 months 16,540 ± 18,066 pg/mL vs. 12 months 13,948 ± 19,329 pg/mL; *p* = 0.048). Interestingly, the decrease in IL-1β (Figure 3B) and IL-6 (Figure 3C) was significantly greater in those patients with worse vitamin D status before supplementation.

In the subgroup of patients in whom peripheral blood immune cells assessment was performed, some subsets of cells exhibited variations in interest (Supplementary Table S4). On the one hand, a significant decrease in the percentage of naïve Treg and activated TCD8 cells was observed. On the other hand, Central Memory Treg, TH1-TH17 populations, and Memory B cells significantly increased over time. Interestingly, the decline in activated CD8 T cells correlated with the IL-1β reduction (R = 0.565; *p* = 0.035, Figure 3D).

## 4. Discussion

This prospective study showed that vitamin D supplementation and micronutrient optimization in patients with decompensated cirrhosis stabilized after hospital discharge improve cognitive function. Importantly, inflammation was found to be linked to the severity of hypovitaminosis D having a negative impact on cognition. Interestingly, patients with more severe vitamin D shortage were the ones with a greater amelioration in neuroinflammatory markers following supplementation.

Cognitive dysfunction in patients with decompensated cirrhosis frequently occurs either as an acute change in mental state or as chronic neuropsychiatric manifestations. The diagnosis of HE implies the exclusion of alternative diagnoses and the presence of compatible symptoms in patients with predisposing conditions [2,33]. Although, in clinical routine, establishing the diagnosis of HE is often not challenging, the difficulty is in attributing a given neurological alteration to a specific cause [33]. In fact, several factors unrelated to liver failure that impact brain function usually coexist in those patients [37]. On the one hand, liver disease etiology such as alcohol [38], viral hepatitis [39] or MASLD [40] may individually affect brain function. Indeed, alcohol was associated with persistent neuropsychological abnormalities following successful liver transplantation [38,41]; attention, working memory and executive function, among others, were found to be altered in patients with hepatitis C, even in those with mild liver disease [39]. Worse cognitive performance was observed in MASLD patients, independent of cardiovascular risk factors [40]. On the other hand, comorbidities such as diabetes, hypertension, aging, hypothyroidism and medications may contribute to cognitive dysfunction [42]. Micronutrient deficiencies also occur in alcoholic and non-alcoholic cirrhosis [43]. Vitamin D deficiency may have a role, giving its pleiotropic extraskeletal functions. Of particular interest, vitamin D shortage is a predisposing risk factor for cognitive impairment [20,21], and it has immunomodulatory properties [17]. In this study performed on cirrhotic patients discharged after decompensation, we observed that hypovitaminosis D was associated with specific worse learning and working memory performance assessed using the Hopkins free recall and WAIS IV letters and number sequencing subtests, respectively (Figure 2). Regarding the cognitive indexes, processing speed and attention were associated with inflammatory response, particularly with IL-1β (Table 3). Similarly, the fall risk assessment tools, Tinnetti balance and Tinnetti score also correlated with IL-1β.

Low-grade cirrhosis-associated immune dysfunction is present in cirrhotic patients without ACLF and is characterized by the production of pro-inflammatory cytokines such as Il-1β, IL-6, TNF-α and INF-γ [7]. Other alterations include TH1 polarization [9,10] and B cell activation [11]. The pathogenesis of low-grade systemic inflammation in decompensated cirrhosis is not well known, although it has been linked to bacterial translocation, the release of damage-associated molecular patterns from injured hepatocytes and progressive loss of immunological tolerance [6]. In this study, in which we evaluated the potential role of several neuroinflammatory markers, IL-1β was found to be involved in cognitive function and fall risk (Table 3). This cytokine is produced by different leucocytes, endothelial cells and epithelial cells and has been associated with acute and chronic inflammation. In the central nervous system, IL-1β is produced by microglia, astrocytes and endothelial cells, and, in physiological conditions, it plays important roles in plasticity, cell growth and transmission [44]. However, in pathological circumstances, IL-1β can act as an inflammatory mediator causing tissue dysfunction [45]. Actually, it was found to be a key piece in sepsis-related cognitive impairment [46] or neurodegenerative diseases contributing to the exacerbation of neuroinflammation [47]. In fact, it has been shown that IL-1β contributes to the impairment of hippocampal-dependent memory in animal models [48].

In addition to the link between IL-1β and cognition, in this study, we found an association between low 25OHD and worse performance on some cognitive tests (Figure 2). These associations are in agreement with previous reports showing that hypovitaminosis D predisposes individuals to different forms of cognitive impairment [20,21,49] and the development of overt and covert HE [24,28], in line with the negative impact of IL-1β on brain function [44,46,47,48,50]. Moreover, we found an improvement in working memory 6 months after nutritional supplementation (Table 2) and amelioration in several neuroinflammatory markers including IL-1β (Table 4). In the subgroup of patients with peripheral immune blood cells assessment, we found a negative correlation between 25OHD and the percentage of TH1 cells at baseline (Figure 3A). Following nutritional supplementation, we observed a significant decrease in the percentage of Treg naive and TCD8 activated (this correlated with the decrease in IL-1β) and an increase in Treg Mcentral, TH1-TH17 population and B memory cells (Supplementary Table S4). The interpretation of these findings may be complex but, on the one hand, it is possible to speculate about the potential impact of hypovitaminosis D enhancing TH1 polarization; on the other hand, we see a shift from an activated phenotype towards a memory phenotype (a decrease in TCD8-activated cells and an increase in B memory cells) with vitamin replacement. These changes could be expected merely because of the natural evolution after acute decompensation. Whether this shift promoting the immune system deactivation is modulated by vitamin D replacement is not clarified here.

Previous in vitro and in vivo studies described the potential anti-inflammatory and tolerogenic effect of vitamin D preventing over-action of the immune system [22]. Indeed, a decrease in pro-inflammatory cytokines following vitamin D supplementation has been previously shown [51]. Our findings support the notion that treating hypovitaminosis D has anti-inflammatory effects on low-grade inflammation and cognitive benefits. The mechanisms are not clarified but may include the direct action of vitamin D on the brain (providing that vitamin D receptor has been identified in brain cells) [52] or indirect mechanisms related to systemic immunomodulatory effects [22,23]. However, a causal association is difficult to establish. We did not find any direct association between cognitive improvement and changes in plasma levels of 25OHD or a decrease in cytokines. Data from clinical trials testing the effect of vitamin D supplementation indeed failed to show solid clinical benefits. This may be in part because of the heterogeneity of the design (vitamin D metabolite used in supplementation, dose, time of supplementation and degree of hypovitaminosis to treat). In this study, our patients achieved mean normal 25OHD following 6 months of supplementation, although six patients continued to show a deficiency and four continued to show an insufficiency. Despite this significant improvement in plasma levels of 25OHD and the amelioration in markers of inflammation, we could not see a clear benefit in the incidence of infections or falls.

This study has several limitations to be considered. The methods used to measure cognitive function may be sensible to re-exposition. Conversely, the chosen cognitive battery includes either a block of trials to practice or alternated versions of tests as available. Additionally, the evaluation was separated by 6 months from the baseline to minimize the learning effects. On the other hand, a control group without hypovitaminosis or no supplementation would be useful to better understand the changes in CAID and cognition in stabilized decompensated cirrhotic patients. However, this group would be difficult to obtain given that decompensated cirrhosis is a population at a high risk of vitamin D deficiency, few subjects would be eligible and patients with vitamin D deficiency could not be maintained without supplementation once identified [19]. Moreover, information regarding vitamin D activity (1,25OHD, vitamin D receptor activation...) and its association with clinical effects would be helpful to better understand the mechanisms behind immunomodulation and cognition. Furthermore, other supplemented micronutrients could have affected the outcomes, although we were not able to find any associations in this study. Finally, the small sample size makes it difficult to have enough statistical power to identify a robust impact on clinical outcomes and also impacts the generalization of these results.

Despite these limitations, we believe that this study provides clinically relevant information. Given that CAID is a hallmark of decompensated cirrhosis, driving poor outcomes, and HE is an inflammatory condition, modulating CAID becomes an extremely attractive approach. Indeed, any strategy able to minimize CAID could abate further decompensation and reduce morbimortality. However, it is also challenging since stimulating or inhibiting the immune system may either predispose individuals to organ damage or increase the risk of infection. Considering that interventions modulating CAID with a favourable risk–benefit profile are currently scarce [6], the extraskeletal functions of vitamin D and its safe profile make this molecule an attractive option. In addition, measuring vitamin D status in clinical practice has already been established, indicating that the contribution to ameliorate CAID and perhaps improve cognition by treating hypovitaminosis D can be easily achieved.

## 5. Conclusions

To summarize, this prospective study shows that vitamin D supplementation safely modulates low-grade inflammation in decompensated cirrhosis with hypovitaminosis D following acute decompensation and provides cognitive benefits. Subjects with more severe vitamin shortages seem to benefit the most. Our results reinforce the current guidelines and recommendations on vitamin D supplementation in cirrhotic patients with hypovitaminosis D.

## Figures and Tables

**Table 1:** Baseline characteristics of the decompensated cirrhotic patients included in the study according to the hospital of inclusion.

	All N = 27	HGUGM N = 14	HSCSP N = 13	*p* Value
DEMOGRAPHICS				
Age (years; mean ± SD)	62.4 ± 11.3	57.57 ± 11.2	67.6 ± 9.2	0.017 *
Gender (male; n, %)	22 (81.5%)	12 (85%)	10 (77%)	0.56
Education (years; mean ± SD)	9.0 ± 4.0	8.0 ± 4.0	10.1 ± 3.8	0.162
ANTHROPOMETRY				
BMI (kg/m^2^; mean ± SD)	27.9 ± 3.6	27.5 ± 3.5	28.3 ± 3.7	0.59
COMORBIDITIES				
Charlson index (mean ± SD)	6.0 ± 1.6	5.9 ± 1.8	6.2 ± 1.5	0.73
CONCOMITANT TREATMENT				
Antidepressants (n, %)	1 (3.7%)	0 (0.0%)	1 (7.7%)	0.290
Benzodiazepines (n, %)	2 (7.4%)	0 (0%)	2 (15.4%)	0.127
Opioids (n, %)	1 (3.7%)	0 (0%)	1 (7.7%)	0.290
Diuretics (n, %)	19 (70.4%)	9 (64.3%)	10 (77%)	0.472
Betablockers (n, %)		7 (50%)	9 (69%)	0.310
CHARACTERISTICS OF CIRRHOSIS				
Alcohol etiology (n, %)	9 (33.3%)	4 (28.6%)	5 (38.5%)	0.90
MELD (mean ± SD)	11.7 ± 4.0	14.0 ± 3.8	9.2 ± 2.5	0.001 *
Esophageal varices (n, %)	21 (77.8%)	11 (78.6%)	10 (77%)	0.918
Ascites (n, %)	24 (88.9%)	14 (100%)	10 (77%)	0.057
SBP (n, %)	7 (25.9%)	5 (36%)	2 (15.4%)	0.228
Non-SBP infection (n, %)	8 (29.6%)	7 (50%)	1 (7.7%)	0.033 *
Variceal bleeding (n, %)	12 (44.4%)	7 (50%)	5 (38.5%)	0.547
Previous OHE (n, %)	9 (33.3%)	6 (42.9%)	3 (23%)	0.276
INFECTION				
Patients with infections within the previous year (n, %)	12 (44.4%)	9 (64%)	3 (23%)	0.054
Number of episodes (median [IQR])	2 [2]	2 [1]	2 [2]	0.864
Hospital admission needed (n, %)	12 (100%)	9 (100%)	3 (100%)	1.00
ICU admission (n, %)	1 (3.7%)	1 (16%)	0 (0%)	0.546
Antibiotics needed (n%)	12 (100%)	9 (100%)	3 (100%)	1.00
DECOMPENSATIONS within the previous year				
Number of hospital admissions within the previous year (median [IQR])	2 [2]	2 [2]	1.5 [1]	0.616
Ascites (n)	25	12	13	0.506
SBP (n)	6	3	3	1
Variceal bleeding (n)	9	4	5	0.823
Overt HE (n)	12	7	5	0.361
LABORATORY TESTS				
Hb (gr/dL, mean ± SD)	11.7 ± 1.6	12.2 ± 1.4	11.3 ± 1.8	0.17
Platelets (1 × 10^9^/L; mean ± SD)	84.3 ± 31.3	83.5 ± 23.73	85.2 ± 38.8	0.889
INR (mean ± SD)	1.3 ± 0.2	1.4 ± 0.2	1.2 ± 0.1	0.006 *
Creatinine (mg/dL; mean ± SD)	0.9 ± 0.2	0.9 ± 0.3	0.8 ± 0.1	0.202
Na (mEq/l; mean ± SD)	138.4 ± 3.9	138.9 ± 4.5	137.9 ± 3.1	0.541
AST (UI/l; mean ± SD)	47.2 ± 44.6	59.4 ± 59.7	34.2 ± 8.9	0.145
ALT (UI/l; mean ± SD)	32.3 ± 34.3	40.7 ± 46.0	23.0 ± 8.6	0.181
GGT (UI/l; mean ± SD)	130.7 ± 109.6	132.0 ± 91.6	129.4 ± 130.0	0.952
ALP (UI/l; mean ± SD)	156.5 ± 64.2	152.8 ± 67.8	160.1 ± 63.0	0.776
Albumin (gr/dL; mean ± SD)	3.4 ± 5.0	3.5 ± 0.6	3.3 ± 0.3	0.370
Ca (mg/dL; mean ± SD)	9.1 ± 0.5	9.2 ± 0.5	8.9 ± 0.6	0.238
Phosphate (mg/dL; mean ± SD)	3.4 ± 0.8	3.6 ± 0.7	3.2 ± 0.8	0.154
Triglycerides (mg/dL, mean ± SD)	86.3 ± 34.3	90.5 ± 37.7	81.8 ± 30.9	0.519
Cholesterol (mg/dL, mean ± SD)	132.6 ± 42.9	138,1 ± 41.2	126.7 ± 45.5	0.500
Prealbumin (mg/dL, mean ± SD)	8.7 ± 4.1	8.1 ± 4.5	9.4 ± 3.5	0.437
Mg (mg/dL; mean ± SD)	1.8 ± 0.2	1.8 ± 0.2	1.8 ± 0.2	0.809
Zn (µg/L; mean ± SD)	56.4 ± 13.5	55.93 ± 13.7	56.9 ± 13.7	0.850
Cu (µg/L; mean ± SD)	89.2 ± 30.6	88.4 ± 31.1	85.6 ± 27.4	0.615
Ferritin (µg/L; mean ± SD)	220.2 ± 274.6	253 ± 333	178 ± 182	0.511
RCP (mg/dL; mean ± SD)	1.0 ± 1.3	1.23 ± 1.5	0.85 ± 1.0	0.457
Procalcitonin (µg/L; median [IQR])	0.09 [0.13]	0.08 [0.13]	0.1 [0.17]	0.151
25OHD (µg/L; mean ± SD)	14.7 ± 8.4	17.74 ± 7.2	11.4 ± 8.7	0.048 *
25OHD sufficient/insufficient/deficient (n)	0/8/19	0/6/8	0/2/11	0.209
PTH (ng/L; mean ± SD)	41.3 ± 20.1	42.7 ± 21.6	39.7 ± 19.1	0.715
Vitamin A (µg/dL; mean ± SD)	17.8 ± 10.9	20.0 ± 9.7	15.6 ± 12.1	0.319
Vitamin E (µg/dL; mean ± SD)	1137.0 ± 440.7	1227.1 ± 388.9	1039.9 ± 487.1	0.279
Vitamin B12 (ng/L; mean ± SD)	701.4 ± 274.6	747.0 ± 288.8	542.0 ± 148.9	0.196
Folate (µg/L; mean ± SD)	10.9 ± 5.6	10.4 ± 5.7	11.6 ± 5.7	0.616

* Denotes statistical significance. BMI: body mass index; MELD: Model End-Stage Liver Disease; SBP: Spontaneous Bacterial Peritonitis; OHE: overt hepatic encephalopathy; Hb: hemoglobin; INR: international normalized ratio; Na sodium; AST: aspartate aminotransferase; ALT: alanine aminotransferase; GGT: gamma glutamyl transferase; ALP: alkaline phosphatase; Ca: calcium; Mg: magnesium; Zn: zinc; Cu: cupper; RCP: reactive C protein, 25OHD: 25-hydroxyvitamin D; PTH: parathyroid hormone.

**Table 2:** Cognitive performance falls and fracture assessment at baseline, 6 and 12 months after inclusion.

		6	12	P	P	P
	Baseline (n = 27)	Months (n = 27)	Months (n = 22)	(6 m vs. Baseline)	(12 m vs. Baseline)	(3 Visits)
COGNITIVE ASSESSMENT						
PHES (score; median [IQR])	0.0 [3]	0.0 [4]	−1 [5]	0.302		0.323
Processing speed (T-score; mean ± SD)	46.5 ± 9.2	48.1 ± 12.1	45.9 ± 10.9	0.051		0.554
Executive (T-score; mean ± SD)	45.6 ± 11.2	49.6 ± 9.8	47.5 ± 9.4	0.070		0.223
Visuomotor (T-score; mean ± SD)	38.7 ± 11.5	39.9 ± 11.3	37.5 ± 10	0.202		0.360
Learning and memory (T-score; mean ± SD)	53.9 ± 11.1	51.9 ± 9.7	50.9 ± 11.6	0.346		0.285
Working memory (T-score; mean ± SD)	46.7 ± 13	50 ± 11	50 ± 12	0.047 *		0.412
Attention (T-score; mean ± SD)	35.6 ± 9.2	35.3 ± 7.5	35.2 ± 7.0	0.627		0.881
Global cognitive function (T-score; mean ± SD)	44 ± 9	45.6 ± 8	43.5 ± 8.4	0.299		0.281
FALLS ASSESSMENT						
Falls (mean ± SD) (events/patient/month)	0.097 ± 0.2 †		0.05 ± 0.07 ‡		0.359	
Tinetti balance (score; mean ± SD)	14.7 ± 1.9	15.1 ± 2.2	14.0 ± 3.0			0.353
Tinetti gait (score; mean ± SD)	11.6 ± 0.9	10.5 ± 3.8	11.1 ± 2.7			0.374
Tinetti total (score; mean ± SD)	26.3 ± 2.7	26.7 ± 3.3	25.2 ± 4.7			0.395
FRACTURES ASSESSMENT						
FRAX major osteoporotic (%; median [IQR])	2.8 [4]		3.1 [4.1]		0.331	
FRAX hip fracture (%; median [IQR])	0.6 [2]		0.6 [1.4]		0.702	

* Denotes statistical significance. † Falls/patient/month during the year before inclusion. ‡ Falls/patient/month during follow-up. PHES: Psychometric Hepatic Encephalopathy Score; FRAX: Fracture Risk assessment tool.

**Table 3:** Baseline correlations between cognitive indexes, 25OHvitD and markers of neuroinflammation (n = 27).

	PHES (Score)	Processing Speed (T-Score)	Executive (T-Score)	Visuomotor (T-Score)	Learning and Memory (T-Score)	Working Memory (T-Score)	Attention (T-Score)	GCF (T-Score)
25OHD (µg/L)	R = 0.06 *p* = 0.77	R = 0.06 *p* = 0.80	R = 0.24 *p* = 0.25	R = 0.06 *p* = 0.78	R = 0.34 *p* = 0.09	R = 0.44 *p* = 0.09	R = 0.16 *p* = 0.51	R = 0.20 *p* = 0.32
CCL2-MCP1 (pg/mL)	R = −0.09 *p* = 0.69	R = −0.09 *p* = 0.68	R = 0.01 *p* = 0.97	R = −0.21 *p* = 0.316	R = −0.29 *p* = 0.12	R = −0.22 *p* = 0.46	R = 0.07 *p* = 0.79	R = −0.20 *p* = 0.32
IL12p70 (pg/mL)	R = −0.13 *p* = 0.57	R = −0.08 *p* = 0.74	R = 0.08 *p* = 0.75	R = −0.08 *p* = 0.73	R = −0.04 *p* = 0.85	R = −0.18 *p* = 0.56	R = 0.02 *p* = 0.93	R = −0.06 *p* = 0.77
IL1 β (pg/mL)	R = −0.42 *p* = 0.05	R = −0.42 * p = 0.04	R = −0.290 *p* = 0.18	R = −0.36 *p* = 0.08	R = −0.06 *p* = 0.78	R = −0.08 *p* = 0.79	R = −0.48 * p = 0.04	R = −0.32 *p* = 0.11
GM-CSF (pg/mL)	R = −0.23 *p* = 0.30	R = −0.18 *p* = 0.40	R = 0.03 *p* = 0.88	R = −0.24 *p* = 0.26	R = −0.02 *p* = 0.92	R = 0.11 *p* = 0.71	R = −0.17 *p* = 0.48	R = −0.15 *p* = 0.46
TNF-α (pg/mL)	R = −0.10 *p* = 0.68	R = −0.25 *p* = 0.24	R = 0.20 *p* = 0.37	R = −0.15 *p* = 0.46	R = −0.13 *p* = 0.52	R = 0.10 *p* = 0.73	R = −0.03 *p* = 0.91	R = −0.12 *p* = 0.58
IL-6 (pg/mL)	R = 0.14 *p* = 0.55	R = −0.06 *p* = 0.77	R = 0.18 *p* = 0.42	R = 0.05 *p* = 0.82	R = −0.15 *p* = 0.47	R = 0.20 *p* = 0.49	R = −0.03 *p* = 0.89	R = −0.01 *p* = 0.97
CX3CL1/ FRACTALKINE (pg/mL)	R = −0.08 *p* = 0.72	R = −0.05 *p* = 0.81	R = −0.03 *p* = 0.91	R = −0.16 *p* = 0.44	R = 0.01 *p* = 0.98	R = 0.05 *p* = 0.87	R = 0.11 *p* = 0.64	R = −0.1 *p* = 0.64
HEPCIDINE (pg/mL)	R = 0.224 *p* = 0.32	R = 0.40 *p* = 0.06	R = −0.06 *p* = 0.77	R = 0.37 *p* = 0.07	R = 0.34 *p* = 0.09	R = 0.29 *p* = 0.31	R = 0.30 *p* = 0.21	R = 0.33 *p* = 0.10
VITDBP (mg/mL)	R = 0.15 *p* = 0.50	R = 0.36 *p* = 0.10	R = −0.28 *p* = 0.20	R = 0.20 *p* = 0.34	R = 0.21 *p* = 0.30	R = −0.28 *p* = 0.33	R = 0.13 *p* = 0.60	R = 0.13 *p* = 0.53

* Denotes statistical significance. 25OHD: 25-hydroxyvitamin D; CCL2-MCP1: Chemokine system CC ligand2-Monocyte chemotactin protein 1; IL12p70: Interleukin 12; IL1 β: Interleukin 1 beta; GM-CSF: Granulocyte Macrophage Colony-Stimulating Factor; TNF-α: Tumour necrosis factor alpha; IL-6: Interleukin 6; CX3CL1/FRACTALKINE: Chemokine (C-X3-C motif) ligand 1; VITDBP: vitamin D binding protein. PHES: Psychometric Hepatic Encephalopathy Score; GCF: global cognitive function.

**Table 4:** Assessment of infections, laboratory tests and inflammatory mediators at baseline, 6 and 12 months after inclusion.

	BASELINE (n = 27)	6 MONTHS (n = 27)	12 MONTHS (n = 22)	P (6 m vs. Baseline)	P (3 Visits)
INFECTIONS					
Episodes/patient/month (mean ± SD)	0.064 ± 0.09 †		0.055 ± 0.1 ‡	0.683	
RCP (mg/dL; mean ± SD)	0.97 ± 1.3	1.0 ± 1.21	0.64 ± 0.2		0.342
Procalcitonin (µg/L; median [IQR])	0.10 [0.13]	0.09 [0.1]	0.095 [0.1]		0.685
MEDICATIONS					
Antidepressants (n%)	1(3.7%)	2(7.4%)	1(4.5%)		1.000
Benzodiazepines (n, %)	2(7.4%)	3(11.1%)	2(9%)		1.000
LABORATORY					
25OHD (µg/L; mean ± SD)	14.6 ± 9.0	37.4 ± 20.4	42.6 ± 18.5		<0.001 *
25OHD sufficient/insufficient/deficient (n)	0/8/19	17/4/6	15/5/2		<0.001 *
PTH (ng/L; mean ± SD)	42 ± 22	32 ± 19	30 ± 11		0.054
Prealbumin (mg/dL; mean ± SD)	8.2 ± 3.7	12.1 ± 5.3	11.4 ± 5.5		0.005 *
Albumin (gr/dL; mean ± SD)	34 ± 4.5	35.7 ± 6.4	36 ± 6.3		0.234
Cholesterol (mg/dL; mean ± SD)	133.2 ± 48	160.7 ± 46.9	172.4 ± 82.5		0.023 *
Vitamin A (µg/dL; mean ± SD)	14.3 ± 9.0	26.7 ± 14.5	27.0 ± 13.1	0.002 *	
Vitamin E (µg/dL; mean ± SD)	1149 ± 499	1304 ± 599	1518 ± 1612		0.288
Ferritin (µg/dL; mean ± SD)	276 ± 304	186 ± 225	173 ± 306		0.256
Hemoglobin (gr/dL; mean ± SD)	11.9 ± 1.6	12.9 ± 2.0	12.8 ± 2.0	0.03 *	0.070
LIVER SCORES					
Child-Pugh (mean ± SD)	6,9 ± 1.5	6.8 ± 1.7	6.8 ± 1.7		0.798
MELD (mean ± SD)	11.1 ± 3.8	11.5 ± 3.3	12.9 ± 4.4		0.053
INFLAMMATION					
CCL2-MCP1 (pg/mL; median [IQR])	270 [149]	256 [114]	284 [169]	0.026 *	0.807
IL12p70 (pg/mL; median [IQR])	2.46 [3.5]	1.98 [2.3]	1.45 [3.7]	0.615	0.920
IL-1β (pg/mL; median [IQR])	0.5 [0.8]	0.3 [0.8]	0.2 [0.9]	0.026 *	0.368
GM-CSF (pg/mL; median [IQR])	2.06 [2.1]	1.8 [1.3]	1.8 [3.6]	0.059	0.807
TNF-α (pg/mL; median [IQR])	12.5 [6.4]	11.3 [4.9]	11.0 [5.5]	0.191	0.607
IL6 (pg/mL; median [IQR])	8.3 [13.8]	7.6 [5.5]	7.5 [6.5]	0.037 *	0.199
CX3CL1/Fractalkine (pg/mL; median [IQR])	1930 [918]	1941 [1414]	1635 [1179]	0.362	0.807
Hepcidine (pg/mL; median [IQR])	17,324 [57,583]	18,445 [24,855]	5715 [16,594]	0.078	0.046 *

* Denotes statistical significance. † Episodes/patient/month during the previous year. ‡ Falls/patient/month during follow-up. RCP: reactive C protein; 25OHD: 25-hydroxyvitamin D; PTH: parathyroid hormone; MELD: Model End-Stage Liver Disease; CCL2-MCP1: Chemokine system CC ligand2-Monocyte chemotactin protein 1; IL12p70: Interleukin 12; IL-1β: Interleukin 1 beta; GM-CSF: Granulocyte Macrophage Colony-Stimulating Factor; TNF-α: Tumour necrosis factor alpha; IL-6: Interleukin 6; CX3CL1/FRACTALKINE: Chemokine (C-X3-C motif) ligand 1.

## Data Availability

The data that support the findings of this study are available from the corresponding author upon reasonable request.

## Supplementary Materials

The following supporting information can be downloaded at https://www.mdpi.com/article/10.3390/nu17020226/s1, Supplementary Paragraph S1: Supplementation of vitamin D according basal 25OHD; Supplementary Table S1: Summary of active nutritional supplementation at each visit; S2: Neurological assessment; S3: Correlation between 25OHD and inflammatory markers (n = 27) and immune cells (n = 14) at the moment of the baseline evaluation; S4: Changes in immune peripheral blood cells overtime. References [53,54,55,56,57,58,59,60,61] are cited in the supplementary materials.
