# Supplementary material for: Vitamin D Supplementation Is Associated with Inflammation Amelioration and Cognitive Improvement in Decompensated Patients with Cirrhosis
Source: Nutrients. 2025 Jan 9;17(2):226. doi: 10.3390/nu17020226 (PMC11768058; doi:10.3390/nu17020226)
Supplement: Supplementary file 1 [file nutrients-17-00226-s001.zip › nutrients-3330554-supplementary.pdf]

SUPLEMENTARY DATA OF PAPER: Vitamin D supplementation is associated with inflammation amelioration and cognitive improvement in decompensated cirrhotic patients.

1. **Supplementary paragraph S1: Supplementation of vitamin D according basal 25OHD.**

1.1. Definition: Vitamin D is a fat-soluble vitamin that exhibits hormonal functions. It can be obtained from a small number of foods, and it is mostly activated in the skin with sun exposure. The most commonly vitamin D metabolite used by clinicians and researchers to indicate vitamin D status is 2OHD. However, taking a single indicator to evaluate these complex interactions has its shortcomings. Besides, when this research was conducted, there was no globally accepted cut-offs for defining vitamin D deficiency and insufficiency. The most common internationally adopted cut-offs for vitamin D deficiency/insufficiency are those proposed by Endocrine Society (ES) and Institute of Medicine (IOM). The practical and clinically accepted cut-off levels for vitamin D adequacy and insufficiency are based on the regulatory mechanisms along the calcium, parathyroid hormone and vitamin D axis.

Table 1. The IOM and ES definitions of vitamin D deficiency (25OHD).

	Sufficiency	Insufficiency	Deficiency
IOM	20-30 ng/mL<br>50-75 nmol/mL	12-20 ng/ml<br>30-50 nmol/L	<12 ng/mL<br><30 nmol/mL
ES	30-100 ng/mL<br>75-250 nmol/mL	20-29 ng/mL<br>50-75 nmol/mL	<20 ng/mL<br><50 nmol/ml

1.2. Recommendations:

The supplementary recommendations are different considering Endocrine Society (ES) and Institute of Medicine (IOM). These differences are mainly based on the population reference for supplementation.

	Reference population	RECOMENDED DOSE	MAXIMUN DAILY DOSE
IOM	Healthy population<br>(low expected benefit)	600-800 UI/day	4000 UI/day
ES	Patients with osteoporosis and other risk conditions<br>(higher objective and doses)	If deficiency:<br>50000 UI/week (8 weeks).<br>Maintenance 1500-2000 UI/day.	4000 UI/day

1.3. Common local clinical practice:

BASELINE VIT D STATUS	INITIAL DOSE	MAINTENANCE	CONTROL	OBJETIVE
INSUFFICIENCY<br>25OHD 20-30 ng/mL	Vitamin D 16000UI/2 weeks	Vitamin D 16000UI /2 weeks	3 months	25OHD<br>30-50 ng/mL<br>Ideally 40ng/mL
DEFICIENCY<br>25OHD <20 ng/mL	Vitamin D 16000UI /1 week	Vitamin D 16000UI /1 week	3 months	25OHD<br>30-50 ng/mL<br>Ideally 40ng/mL

a) Dose and maintenance were individualized in patients with special characteristics such as severe deficiency, malabsorption or suboptimal response.
b) Expected increases in serum 25OHD according to supplementation:
100 UI/d increase serum 25OHD 1-2 ng/mL .
1000UI/d increase serum 25OHD 10-20 ng/mL.
2000UI/d increase serum 20-40 ng/mL.
c) Given that plasma measurements are not perfect, the plasma 25OHD target following supplementation is 40 ng/mL (which ensures adequate vitamin repletion remaining within the normal range).
d) Calcium, Phosphate and 25OHD were monitored during the study.
e) Dose adjustments during follow-up: :
   - If 25OHD < 60 ng/mL: Keep same dose (16000UI /1 week if baseline deficiency and 16000UI/2 weeks if insufficiency)
   - IF5OHD ≥ 60 ng/mL: decrease dose to half of initial dose (16000UI /2 weeks if deficiency and 16000UI/4 weeks if insufficiency).

2. Supplementary table S1: Summary of active nutritional supplementation at each visit.

	Baseline	6 months	12 months	P
Vitamin A (N, %)	12 (44.44%)	16 (59.26%)	16 (72.72%)	0.1347
Vitamin E (N, %)	2 (7.41%)	2 (7.41%)	2 (9.09%)	0.9700
Vitamin D (N, %)	**0 (0%)**	**24/27 (88.8%)**	**21/22 (95.45%)**	**<0.0001**
Vitamin K (N, %)	2 (7.41%)	2 (7.41%)	1 (4.54%)	0.9011
Vitamin B12 (N, %)	2 (7.41%)	0	1 (4.54%)	0.3710
Folic acid (N, %)	3 (11.11%)	4 (14.81%)	1 (4.54%)	0.5034
Calcium (N, %)	2 (7.41%)	2 (7.41%)	2 (9.09%)	0.9700
Magnesium (N, %)	3 (11.11%)	2 (7.41%)	1 (4.54%)	0.6934
Iron (N, %)	5 (18.52%)	6 (22.22%)	3 (13.63%)	0.7427
Hypercaloric oral nutritional supplement (N, %)	2 (7.41%)	2 (7.41%)	1 (4.54%)	0.9011

Comment: At hospital discharge, the identified nutritional deficiencies were supplemented according to local practice. Additionally, if the patient was eligible, then he/she was cited within the 1-6 weeks after discharge for baseline assessment and vitamin D supplementation. This may explain the fact that at the moment of the baseline evaluation 44% of patients were receiving vitamin A, 18% were receiving iron, 11% were on folic acid supplementation and 11% were receiving magnesium among others.

As expected, a significant change in prescription was observed in vitamin D supplementation, and no other statistically significant changes were observed for other micronutrients. Of note, 59% and 72% of patients were receiving vitamin A supplementation at 6 months and12 months respectively.

3. **Supplementary table S2: Neurological assessment.**

Cognitive Domain	Test	Characteristics of the test
Visuo-motor abilities	**Grooved Pegboard**	A visual-motor coordination test, consisting of a small board containing slotted holes angled in different directions. Each peg has a ridge along one side requiring it to be rotated into position for correct insertion. The score is the time to completion in seconds with the dominant and non-dominant hand.
	**WAIS IV Block Design subtest**	Block design is a subtest that is administered as part of Wechsler Adult Intelligence Scale-fourth edition[1] targeting visual-spatial organization and non-verbal problem solving, and motor skills. The patients use hand movements to rearrange blocks with surfaces of solid red, surfaces of solid white, and surfaces that are half red and half white to replicate a previously presented pattern. Norms are available for Spain [2]
	**THE PHES subtest** serial dotting test	A test of fine motor activity. The patient has to insert a dot inside the circles which are disposed in lines. Time is measured[3].
	**The PHES subtest** line drawing test	A test that examines vuoespatial coordination. The patient paints a continuous line inside the space limited by two given parallel lines. Paper shouldn’t be moved and given lines can´t be crossed. Time is measured[3].
Executive function	**THE PHES subtest** number connection test A	The patient will have to join the number as fast as he can in the same order as if he will be counting. He has to choose always the shortest path. A test measuring visual search speed, mental flexibility and executive functioning[3].
	**THE PHES subtest** number connection test B	The patient must connect numbers and letters orderly, alternating them; This means from 1 to A, from 2 to B and so on, until the end. He should perform this test as quickly as possible. A test influenced by resistance to distraction and cognitive flexibility (set-shifting) useful to assess executive functioning[3].
	**Stroop Test interference score**	Stroop interference score is used to assess cognitive flexibility as the ability to inhibit cognitive interference that occurs when the processing of a stimulus feature (i.e. colour) impedes the simultaneous processing of another attribute of the same stimulus (i.e. reading)[4-7]
Processing speed	**The PHES subtest** digit symbol test (DST),	The patient will see nine squares, each showing a number at the top and a symbol at the bottom. Each symbol is related to a number. The patient must fill in the missing symbols in the empty squares. Evaluates visual scanning and mental processing speed[3].

Attention	**Stroop Colour and Word scores from the Stroop test**	The patient has to name a colour or read a word as a measure of selective attention capacity and processing speed ability[4-7].
***Working memory*:**	**WAIS IV Letter-Number sequencing subtest**	A subtest that is administered as part of WAIS-IV[1]measuring working memory by means of randomly ordered sequences of numbers and letters where the participant is asked to respond by stating the numbers first (ordered from the smallest to the largest number) and the letters second (in alphabetical order) [2].
***Learning and memory*:**	**Hopkins verbal learning test**	It is a test assessing verbal learning and memory by means of a three-trial list learning and free recall task comprising 12 words, 4 words from each of three semantic categories with six equivalent alternate forms for longitudinal studies[8-9].

4. **Supplementary table S3. Correlation between 25OHD and inflammatory markers (n=27) and immune cells (n=14) at the moment of the baseline evaluation**

	Pearson or Spearman	p
NEUROINFLAMMATORY MARKERS (N=27)		
Ccl-2/mcp-1 (pg/mL)	-0.211	0.300
IL-12p70 (pg/mL)	0.049	0.821
IL-1β(pg/mL)	-0.220	0.280
GM-CSF (pg/mL)	0.016	0.940
TNF-α (pg/mL)	-0.148	0.469
IL-6 (pg/mL)	-0.174	0.396
Cx3CL1/Fractalkine (pg/mL)	0.001	0.997
Hepcidine (pg/mL)	-0.111	0.589
VitDBP(mg/mL)	0.001	0.997
PERIPHERAL BLOOD IMMUNE CELLS (%; N=14)		
Central Memory Treg%	-0.247	0.394
Lymphocytes%	0.354	0.215
CD3%	-0.030	0.919
CD4 T cells%	-0.201	0.490
Naïve CD4 T cells%	-0-095	0.747
Central Memory CD4 T cells%	0.044	0.881
Effector Memory CD4 T cells%	0.386	0.173
TEMRA CD4 T cells%	-0.162	0.580
Activated CD4 T cells%	-0.049	0.869
Treg%	-0.206	0.480
Naïve Treg%	0.146	0.619
Effector Memory Treg%	0.345	0.228
TEMRA Treg%	0.04	0.891
Activated Treg%	-0.465	0.094
TH0 %	0.076	0.795
TH1 %	**-0.543**	**0.045**
TH2 %	0.002	0.994
TH17 %	-0.234	0.422
CD8 T cells%	-0.349	0.221
B cells%	-0.053	0.857
BregI%	0.132	0.653
BregII%	0.234	0.422
NKT%	-0.039	0.894
Neutrophils%	0.350	0.220
Eosinophils%	-0.035	0.906
Basophils %	0.289	0.316
Monocytes %	-0.012	0.969
Dendritic cells%	0.169	0.564
MDC%	0.09	0.759
PDC%	0.284	0.324
CD8 T cells%	0.432	0.123

5. **Supplementary table S4: Changes in immune peripheral blood cells overtime.**

	BASELINE	6 MONTHS	12 MONTHS	P (baseline vs 6 months)	P (3 visits)
**RELATIVE NUMBERS Median (p25-p75)**					
Central Memory Treg%	**71 (59-84)**	**76 (68-84)**	**79 (67-85)**	**0.046***	**0.011***
Lymphocytes%	28.9 (25-33)	24 (21-30)	23(17-30)	0.177	0.093
CD3%	72 (61-79)	74.2 (68-79)	71 (65-77)	0.594	0.882
CD4 T cells%	43 (26-51)	49 (38-53)	46 (36-53)	0.730	0.882
Naïve CD4 T cells%	21 (10-34)	21 (8-35)	21 (9-20)	0.074	0.197
Central Memory CD4 T cells%	57 (45-68)	54 (45-69	55 (49-70)	0.826	0.223
Effector Memory CD4 T cells%	12 (7-19)	15 (8-20)	10 (6-18)	0.286	0.325
TEMRA CD4 T cells%	1.6 (0.14-5.78)	1.7 (0.16-3.93	1.3 (0.09-5.3)	0.875	0.542
Activated CD4 T cells%	4 (3.25-4.6)	4.5 (2.5-9.5)	3.4 (3.2-5.4)	0.778	0.417
Treg%	7(4.6-10.8)	7(4.7-8.5)	5.2 (4.4-8.1)	0.397	0.882
Naïve Treg%	**13.7 (9.3-28.8)**	**12.2 (5.7-24.7)**	**9.6 (5.3-23.6)**	**0.041***	**0.034***
Effector Treg%	3 (1.4-6)	2.4 (2-4.4)	2.1 (1.2-3.6)	0.683	0.687
TEMRA Treg%	0	0	0	0.888	0.529
Activated Treg%	15 (9.6-18.5)	16.6 (14.6-19)	16.1 (11.6-22.6)	0.925	0.882
TH0%	31 (20-40)	26 (24-35)	29 (14-38)	0.510	0.325
TH1%	11 (5-15)	15 (12-19)	16 (9-19)	0.124	0.135
TH2%	7 (4.8-9.8)	5.7 (5-7)	5.5 (5.1-6.0)	0.975	0.687
TH17%	11.2(6.8-12)	7.2 (4.2-9.5)	7.8 (6.3-14)	0.140	0.135
TH1-TH17%	**15 (3.7-17.6)**	**17.4 (9-19.8)**	**19.8 (15.2-24.5)**	0.245	**0.044***
CD8 T cells%	18.6	18.8	18.3	0.300	0.607
Naïve CD8 T cells%	22.6	18	17.4	0.433	0.882
Central Memory CD8 T cells%	15.3	17.8	17.3	0.096	0.417
Effector Memory CD8 T cells%	7.2	8.7	9.3	0.510	0.607
Effector Memory Type 1 CD8 T cells%	66.5	53.9	60	0.103	0.223
Effector Memory Type 2 CD8 T cells%	31.5	44.4	38.7	0.124	0.223
TEMRA CD8 T cells%	37	35.7	31.8	0.875	0.417
CD8 Treg%	50.8	51	49	0.510	0.417
Activated CD8 T cells%	**4.3 (2.2-8)**	**3.9 (2.3-7.13)**	**2.6 (1.2-6.2)**	0.382	**0.03***
B cells%	5.3	9.8	9.9	0.084	0.417
Naïve B cells%	9.4 (2.7-72)	65.8 (48-72)	55 (33-80)	0.109	0.197
Memory B cells%	**1.8 (0.4-4.6)**	**7.5 (2.9-11)**	**8 (4.6-11.4)**	**0.035***	**0.005***

Memory Switch B cells%	11	12	19.4	0.875	0.325
CD27 Memory B cells%	42(6.6-68)	16.5 (9-22)	15 (8-30)	0.084	0.135
BregI%	1.9	2	1.4	0.778	1.000
BregII%	4.3	2.6	4.2	0.433	0.417
PLASMABLASTS%	1.7	1.1	1.9	0.124	0.607
NKT%	1.3	0.6	0.02	0.272	0.159
Granulocytes%	53.8 (44-57)	50 (44-64)	59 (45-67)	0.925	0.223
Neutrophils%	90	89	89	0.730	0.607
Eosinophils%	7.7	9.2	9.4	0.221	0.798
Basophils%	0.75	0.71	0.75	0.638	0.687
Monocytes%	8.9	9.5	8.6	0.363	0.687
Dendritic cells%	2.2 (1.7-3.4)	2.9 (0.7-5.3)	3.7 (1.5-5.7)	0.079	0.197

***Statistically significant**

REFERENCES:

1. Wechsler, David. 2008. Wechsler Adult Intelligence Scale--Fourth Edition., . doi:10.1037/t15169-000.
2. https://www.pearsonassessments.com/professional-assessments/products/spanish-assessments-english.html (accessed on 2 November 2024).
3. Gómez, Manuel Romero, Córdoba, Juan, Jover, Rodrigo, del Olmo, Juan, Fernández, Ana, Flavià, Montse, Compañy, Luis, Poveda, María José, and Vicente Felipo. 2006. Tablas de normalidad de la población española para los tests psicométricos utilizados en el diagnóstico de la encefalopatía hepática mínima. *Medicina Clinica* 127: 246–249. doi:10.1157/13091264.
4. Stroop, J.R. 1992. Studies of interference in serial verbal reactions. *Journal of Experimental Psychology: General* 121: 15-23. doi: 10.1037/0096-3445.121.1.15
5. Golden, C.J. 1978. Stroop Color and Word Test: A Manual for Clinical and Experimental Uses. Chicago, Illinois: Skoelting: 1-32.
6. Rognoni, T., Casals-Coll, M., Sánchez-Benavides, G., Quintana, M., Manero, R.M., Calvo, L., Palomo, R., Aranciva, F., Tamayo, F., and J. Peña-Casanova. 2013. Estudios normativos españoles en población adulta joven (proyecto NEURONORMA jóvenes): normas para las pruebas Stroop Color-Word Interference Test y Tower of London-Drexel University. *Neurología* 28: 73–80. doi:10.1016/j.nrl.2012.02.009.
7. Peña-Casanova, Jordi, Quiñones-Úbeda, Sonia, Gramunt-Fombuena, Nina, Quintana, María, Aguilar, Miquel, Molinuevo, José Luis, Serradell, Mónica, Robles, Alfredo, Barquero, María Sagrario, Payno, Maria, and et al. 2009. Spanish Multicenter Normative Studies (NEURONORMA Project): Norms for the Stroop Color-Word Interference Test and the Tower of London-Drexel. *Archives of Clinical Neuropsychology* 24: 413–429. doi:10.1093/arclin/acp043.
8. Brandt, Jason. 1991. The hopkins verbal learning test: Development of a new memory test with six equivalent forms. *The Clinical Neuropsychologist* 5: 125–142. doi:10.1080/13854049108403297
9. Atxukarro, Oihane Sáez, Sáez, Rocio del Pino, Lasa, Javier Peña, Schretlen, David J., Bilbao, Naroa Ibarretxe, and Natalia Ojeda del Pozo. 2021. Test de aprendizaje verbal de Hopkins revisado: normalización y estandarización de la prueba en población española. *Revista De Neurologia* 72: 35–42. doi:10.33588/rn.7202.2020412.
